# CD56^dim^CD16^dim^ Natural Killer (NK) Cells: The Forgotten Population

**DOI:** 10.1097/HS9.0000000000000348

**Published:** 2020-04-01

**Authors:** Jacques Zimmer

**Affiliations:** Department of Infection and Immunity, Luxembourg Institute of Health, 29 rue Henri Koch, L-4354 Esch-sur-Alzette, Luxembourg

I read with great interest the recent publication by Hofland et al in *HemaSphere*^1^ about the activation of natural killer (NK) cells in chronic lymphocytic leukemia (CLL) in an in vitro model. The authors nicely demonstrate that the functional properties of NK cells (degranulation of cytolytic molecules such as granzyme B, as well as cytotoxicity and IFN-γ production) can be efficiently stimulated via the FcγRIIIa receptor CD16a, whereas natural cytotoxicity via NKG2D is negatively affected. Furthermore, the percentage of “mature” (NKG2C^+^CD57^+^ILT2^+^) cells is significantly higher in CLL patients than in healthy controls. In contrast, the inhibitory receptor KLRG1, considered as a senescence indicator,^2^ has a similar expression in both groups. Further NK cell subset markers, such as CD2, FcεRγ, and the transcription factors Eomes, T-bet, PLZF, could have helped to classify these NK cells in additional categories (conventional, adaptive,…). Nevertheless, the fact that NK cells from CLL patients are fully functional as far as CD16 stimulation is concerned (ie, target cell killing via antibody-dependent cellular cytotoxicity or ADCC), is convincingly shown in this paper and opens interesting therapeutic perspectives.

**Figure 1:**
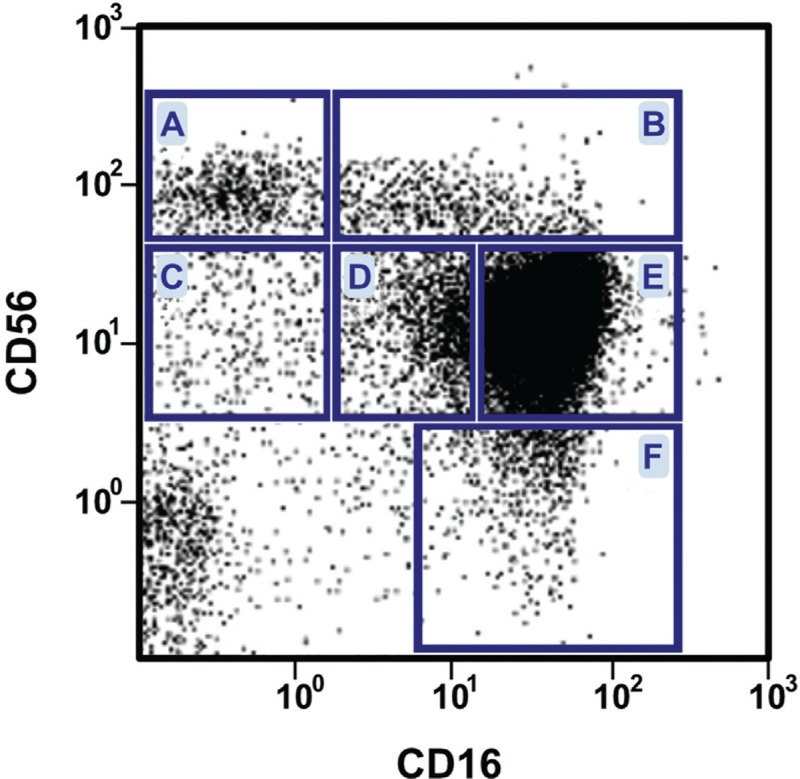
**The six NK cell subpopulations in human peripheral blood**. Dot plot of a flow cytometry experiment after staining of peripheral blood mononuclear cells with fluorescent anti-CD16 (x axis) and anti-CD56 (y axis) antibodies. Monocytes (CD14^+^), B (CD19^+^) and T (CD3^+^) lymphocytes are gated out. The CD56^dim^CD16^dim^ subset corresponds to population ‘D’. The figure was first released in Reference 4 as Figure 1A, and re-use is permitted based on the fact that this work was published as an open-access article under the terms of the Creative Commons Attribution License (CC BY).

The major reason for this comment resides, however, in the gating strategy that the authors applied to the peripheral blood mononuclear cells to end up with three different NK cell populations: CD56^bright^CD16^−^, CD56^dim^CD16^+^ and CD56^-^CD16^+^ (populations A, E, and F in Fig. 1, respectively). Although this matches the subset nomenclature in many other papers, three additional subpopulations are excluded from the analysis by the authors, namely CD56^bright^CD16^dim^, CD56^dim^CD16^-^ and CD56^dim^CD16^dim^,^3–5^ (populations B, C and D, in Fig. 1, respectively), which are likewise all clearly visible in the representative dot plot shown at the upper right part of Suppl. Figure 4A of the Hofland paper.^1^ The CD56^dim^CD16^dim^ subset, which we described in 2017,^4^ appears in the Hofland manuscript^1^ as a substantial part of the total NK cell population, located immediately at the left side of the major CD56^dim^CD16^bright^ cells, which are called here CD56^dim^CD16^+^.

All these remarks are not just related to semantic details. We have shown that the CD56^dim^CD16^dim^ population, clearly apparent in many published dot plots of CD16 *versus* CD56 expression,^6–8^ displays phenotypic differences with CD56^dim^CD16^bright^ cells and likely represents a more immature precursor of the latter. Arguments in favor of this claim are (i) a significantly higher percentage of NKG2A^+^ and CD27^+^ cells opposed to a lower percentage of KIR^+^ NK cells, and (ii) a significantly lower percentage of CD57+ and CD62L+ cells within the CD56^dim^CD16^dim^ subset compared to its CD56^dim^CD16^bright^ counterpart.^4^

The CD56^dim^CD16^-^ NK cells have been described extensively by Stabile et al,^6^ although this group calls the subset CD56^dim^CD16^dim^, which is in fact not exactly right. In addition, Béziat et al^9^ have characterized the CD56^bright^CD16^dim^ cells as yet another subtype and developmental intermediate of peripheral blood NK cells. Finally, our data have been confirmed by other authors,^10^ who also noticed that a low density of expression of CD16 is correlated with further phenotypic and functional differences to the CD56^dim^CD16^bright^ NK cells. Overall, most groups detect the CD56^dim^CD16^dim^ subset, but they either simply gate it out, as Hofland et al,^1^ or they integrate it into the CD56^dim^CD16^bright^ population, despite the obvious fact that the CD56^dim^CD16^dim^ cells express lower levels of CD16.

Thus, the information that could be gathered by including the CD56^dim^CD16^dim^ population into the analysis and by comparing it with the other NK cell subsets, is simply and regrettably lost. Due to the complexity of these different subset names, it could already be helpful if every research team working on human NK cells would follow the nomenclature used by the leaders in the field, which is still valid.^3^
